# Association of the C-reactive protein-triglyceride glucose index with cardiovascular disease and mortality in the general US population: A NHANES study

**DOI:** 10.1097/MD.0000000000049586

**Published:** 2026-07-10

**Authors:** Zhenze Yu, Zihan Zhao, MingZhuang Sun, Yalei Han

**Affiliations:** a Cardiac Department, Aerospace Center Hospital, Beijing, China; b Cardiac Department, Peking University Aerospace School of Clinical Medicine, Beijing, China; c Interventional Center of Valvular Heart Disease, Beijing Anzhen Hospital, Capital Medical University, Beijing, China.

**Keywords:** all-cause mortality, C-reactive protein-triglyceride glucose index (CTI), cardiovascular disease, insulin resistance, NHANES

## Abstract

Cardiovascular disease (CVD) remains a leading global cause of mortality, with metabolic and inflammatory dysregulation playing pivotal roles. The C-reactive protein-triglyceride glucose index (CTI), a novel biomarker integrating inflammation (CRP) and insulin resistance (triglyceride-glucose index), has shown promise in predicting adverse outcomes, but its associations with CVD and mortality in the general population remain unclear. The aim of this study is to explore the association of the C-reactive protein-triglyceride glucose index with cardiovascular disease and mortality. This study analyzed data from 8720 adults in the National Health and Nutrition Examination Survey (NHANES) 2001–2010. CTI was calculated as CTI 0.412 × ln(CRP [mg/L]) + [ln(TG [mg/dL] × FPG [mg/dL])]/2. Multivariable logistic regression examined the association between CTI tertiles (T1–T3) and CVD, and Cox regression assessed the relationship between CTI tertiles and cardiovascular and all-cause mortality, with adjustment for demographic, lifestyle, and clinical factors. Higher CTI tertiles were correlated with worse metabolic profiles and higher prevalence of hypertension and diabetes (*P* < .001). After full adjustment, the highest CTI tertile (T3) was associated with increased CVD risk (odds ratio = 1.50, 95% confidence interval [CI]: 1.18–1.92, *P* = .004), cardiovascular mortality (hazard ratio = 1.54, 95% CI: 1.06–2.25, *P* = .024), and all-cause mortality (hazard ratio = 1.53, 95% CI: 1.29–1.89, *P* < .001). Restricted cubic spline analysis revealed a nonlinear relationship between CTI and *cardiovascular* mortality, *as well as* all-cause *mortality*. Higher CTI is associated with prevalent CVD and independently predicts increased cardiovascular and all-cause mortality.

## 1. Introduction

Cardiovascular disease (CVD) is one of the leading causes of global mortality and disability, with a complex pathogenesis involving multiple risk factors such as elevated low-density lipoprotein cholesterol (LDL-C), hypertension, smoking, and high body mass index (BMI).^[1]^ Dysglycemia, dyslipidemia, and inflammation are pivotal pathophysiological factors in the initiation and progression of CVD.^[2]^ Recent studies have focused on developing integrated biomarkers that comprehensively evaluate these pathological processes to improve CVD risk prediction accuracy. Previous research predominantly investigated individual metabolic or inflammatory markers – such as the triglyceride-glucose (TyG) index, neutrophil-to-lymphocyte ratio, or triglyceride to high-density lipoprotein cholesterol ratio (TG/HDL-C) ratio – yet failed to provide a holistic assessment combining metabolic and inflammatory indicators.^[3,4]^

The TyG index, a simple and reliable surrogate marker of insulin resistance, has been consistently associated with an increased risk of CVD in multiple epidemiological studies.^[5-7]^ C-reactive protein (CRP), a well-established biomarker of systemic inflammation, has been extensively documented to correlate with CVD risk in numerous epidemiological and clinical studies.^[8,9]^

The C-reactive protein-triglyceride glucose index (CTI), a novel composite biomarker integrating inflammatory and metabolic dysregulation markers. Some evidence demonstrates significant associations between elevated CTI levels and adverse clinical outcomes, including coronary artery disease and cancer prognosis.^[10,11]^ However, the associations of CTI with CVD, cardiovascular mortality, and all-cause mortality require further validation. Using large-scale National Health and Nutrition Examination Survey (NHANES) data, this study aim to explore cross-sectional CTI–CVD associations and longitudinal CTI–mortality relationships.

## 2. Materials and methods

### 2.1. Data source

The study utilized data from the NHANES, conducted by the Centers for Disease Control and Prevention (CDC). NHANES employs a complex, multistage probability sampling design to obtain nationally representative health data from the US civilian noninstitutionalized population.

### 2.2. Study population

**Figure 1.**
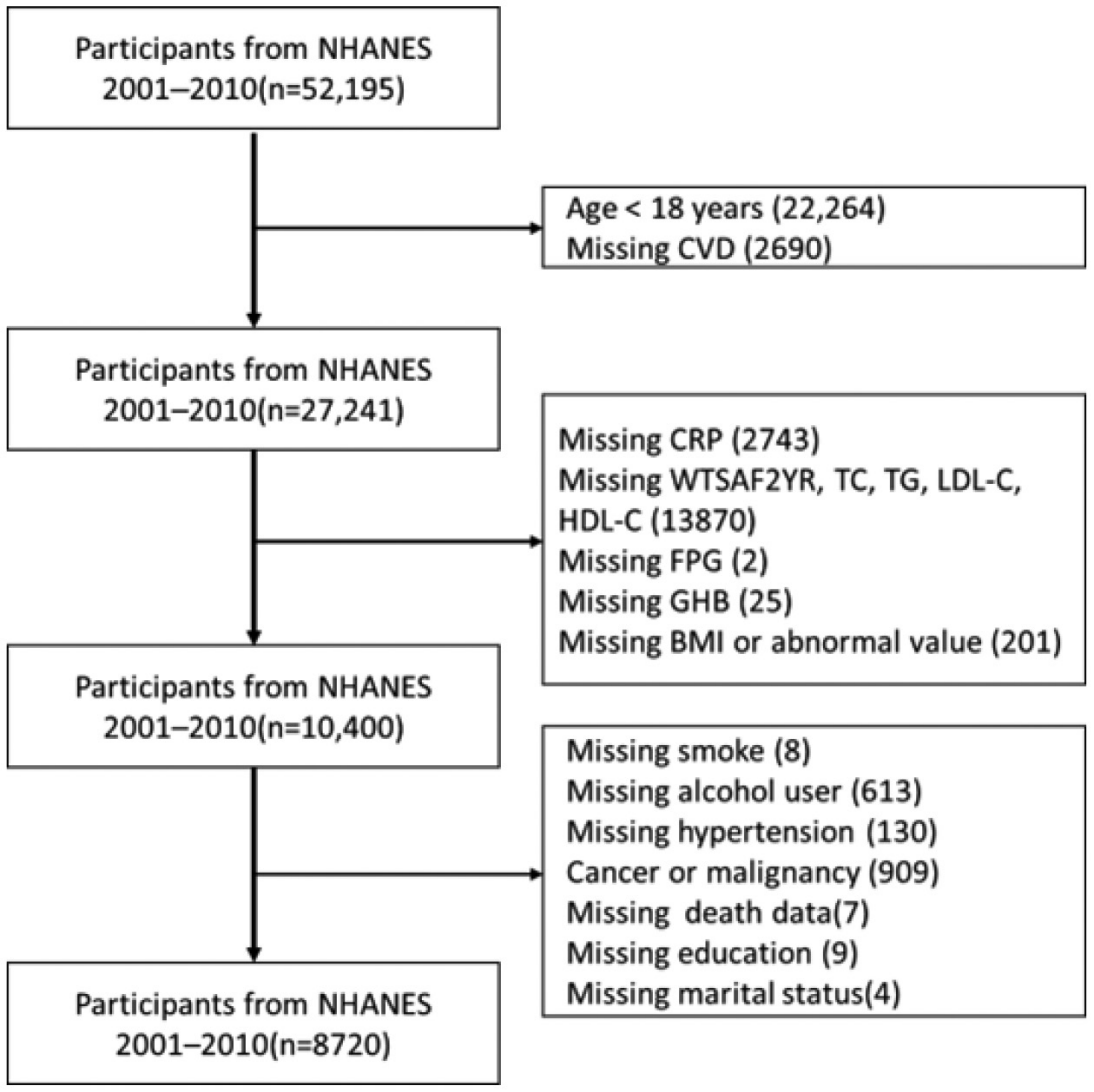
Flowchart of patient selection. From 52,195 NHANES participants (2001–2010), 8720 were included after exclusions for age, missing data, cancer, and other criteria (see abbreviations). BMI = body mass index, CRP = C-reactive protein, CVD = cardiovascular disease, GHB = glycosylated hemoglobin, HDL-C = high-density lipoprotein cholesterol, LDL-C = low-density lipoprotein cholesterol, NHANES = National Health and Nutrition Examination Survey, TC = total cholesterol, TG = triglyceride, WTSAF2YR = Fasting Subsample 2 Year MEC (Mobile Exam Center) Weight.

The study analyzed data from 52,195 participants in NHANES 2001–2010. We excluded 22,264 individuals aged <18 years, followed by 2690 with missing cardiovascular disease data, 18,521 with missing sampling weights, incomplete biomarkers (CRP, lipids, or glycated hemoglobin [HbA1c]), missing covariates (smoking, alcohol use) and cancer or malignancy. One outlier with implausible values was further excluded. The final analytical sample of 8720 participants represents a nationally representative adult population after these exclusions (Fig. 1).

### 2.3. Covariate definitions

Smoking status and alcohol consumption were assessed by self-report, with smoking defined as having smoked ≥100 cigarettes in lifetime and alcohol use as consuming ≥12 alcoholic drinks per year. Hypertension was defined as systolic blood pressure ≥140 mm Hg and/or diastolic blood pressure ≥90 mm Hg, physician-diagnosed hypertension, or current use of antihypertensive medication. Glycemic status was categorized as: normal glucose (fasting glucose <6.1 mmol/L and 2-hour oral glucose tolerance test (OGTT) glucose <7.8 mmol/L and HbA1c <5.7%), prediabetes (fasting glucose 6.1−6.9 mmol/L or 2-hour OGTT glucose 7.8−11.0 mmol/L or HbA1c 5.7−6.4%), or diabetes (fasting glucose ≥7.0 mmol/L or 2-hour OGTT glucose ≥11.1 mmol/L or HbA1c ≥6.5%, physician-diagnosed diabetes, or current use of glucose-lowering medication/insulin).

### 2.4. CTI, CVD, and mortality outcomes

The CTI was calculated using the following formula: CTI = 0.412 × ln(CRP [mg/L]) + [ln(TG [mg/dL] × FPG [mg/dL])]/2. The determination of CVD status in NHANES was based on self-reported physician diagnoses from the Medical Conditions Questionnaire, including congestive heart failure (MCQ160b), coronary heart disease (CHD; MCQ160c), angina pectoris (MCQ160d), myocardial infarction (MCQ160e), and stroke (MCQ160f). Mortality data were obtained through linkage with the National Death Index (NDI) by the National Center for Health Statistics (NCHS), with follow-up through December 31, 2019.

### 2.5. Statistical analysis

Continuous variables were expressed as mean ± SD or median [IQR], categorical as n (%). Group differences were tested using ANOVA/Kruskal–Wallis or χ^2^ tests.

Multivariable logistic regression models were used to evaluate the association between CTI and CVD risk. Three models were constructed: model 1 (unadjusted); model 2 adjusted for demographic and lifestyle factors (age, sex, race, education level, marital status, smoking status, alcohol consumption, and BMI) and model 3 additionally adjusted for clinical biomarkers (LDL-C, hypertension status).

Cumulative survival curves were used to assess differences in cumulative survival for CVD mortality and all-cause mortality across CTI tertiles (T1, T2, T3). Cox survival regression models were employed to examine the association between CTI and mortality risk, with sequential adjustment for confounding factors (unadjusted, partially adjusted, and fully adjusted models). Restricted cubic spline analysis was performed to explore potential nonlinear relationships between CTI and both CVD mortality and all-cause mortality. A 2-sided significance level of α = 0.05 was applied for all statistical tests. All analyses were conducted using R software (version 4.3.1; Lucent Technologies).

## 3. Results

### 3.1. Baseline characteristics

This study analyzed 8720 participants stratified by CTI tertiles (T1–T3). The mean CTI values increased significantly from 7.01 in T1 to 8.95 in T3. Higher CTI tertiles showed progressively worse metabolic profiles, including elevated glucose levels, lipids, and BMI. Clinically, hypertension prevalence doubled (24.8–50.6%) and diabetes rates increased 5-fold (5.3–27.8%) across tertiles. Mortality outcomes showed particularly strong associations, with cardiovascular mortality tripling (2.7–6.5%) and all-cause mortality more than doubling (10.4–21.8%) from T1 to T3. All comparisons were statistically significant (*P* < .001; Table 1).

**Table 1:** Baseline characteristics of the all participants by CTI tertiles.

	Overall	T1	T2	T3	*P*-value
N	8720	2907	2907	2906	
CTI	7.99 ± 0.88	7.01 ± 0.48	8.02 ± 0.22	8.95 ± 0.44	**<.001**
TyG	8.63 [8.24, 9.05]	8.14 [7.89, 8.40]	8.64 [8.39, 8.90]	9.16 [8.88, 9.47]	**<.001**
Age	46.00 [33.00, 62.00]	40.00 [28.00, 54.00]	49.00 [35.00, 65.00]	51.00 [36.00, 64.00]	**<.001**
Sex (male %)	4245 (48.7)	1445 (49.7)	1523 (52.4)	1277 (43.9)	**<.001**
Race (%)					**<.001**
Mexican American	1830 (21.0)	480 (16.5)	598 (20.6)	752 (25.9)	
Other Hispanic	631 (7.2)	198 (6.8)	217 (7.5)	216 (7.4)	
Non-Hispanic White	4256 (48.8)	1429 (49.2)	1438 (49.5)	1389 (47.8)	
Non-Hispanic Black	1654 (19.0)	656 (22.6)	540 (18.6)	458 (15.8)	
Other race – including multiracial	349 (4.0)	144 (5.0)	114 (3.9)	91 (3.1)	
Education (%)					**<.001**
Middle school or lower	2451 (28.1)	621 (21.4)	842 (29.0)	988 (34.0)	
High school	2068 (23.7)	637 (21.9)	699 (24.0)	732 (25.2)	
College or more	4201 (48.2)	1649 (56.7)	1366 (47.0)	1186 (40.8)	
Marital (%)					**<.001**
Married	4777 (54.8)	1498 (51.5)	1650 (56.8)	1629 (56.1)	
Divorce	822 (9.4)	220 (7.6)	290 (10.0)	312 (10.7)	
Other	3121 (35.8)	1189 (40.9)	967 (33.3)	965 (33.2)	
FPG (mmol/L)	5.44 [5.05, 5.94]	5.22 [4.88, 5.55]	5.46 [5.11, 5.88]	5.77 [5.27, 6.61]	**<.001**
GHB	5.40 [5.20, 5.70]	5.30 [5.00, 5.50]	5.40 [5.20, 5.70]	5.60 [5.30, 6.10]	**<.001**
TC (mg/dL)	195.00 [169.00, 223.00]	182.00 [159.00, 208.00]	196.00 [171.00, 223.00]	206.00 [180.00, 236.00]	**<.001**
LDL-C (mg/dL)	115.00 [93.00, 139.00]	106.00 [87.00, 129.00]	119.00 [95.00, 142.00]	122.00 [98.00, 146.00]	**<.001**
HDL-C (mg/dL)	52.00 [43.00, 64.00]	59.00 [49.00, 70.00]	51.00 [43.00, 61.50]	45.00 [39.00, 55.00]	**<.001**
TG (mg/dL)	111.00 [79.00, 162.00]	73.00 [58.00, 93.00]	113.00 [89.00, 143.00]	177.00 [135.00, 231.75]	**<.001**
BMI (kg/m^2^)	27.73 [24.28, 31.88]	24.75 [22.11, 27.92]	28.00 [24.88, 31.57]	30.89 [27.27, 35.76]	**<.001**
Smoke (%)	4057 (46.5)	1207 (41.5)	1370 (47.1)	1480 (50.9)	**<.001**
Alcohol (%)	6166 (70.7)	2172 (74.7)	2056 (70.7)	1938 (66.7)	**<.001**
Hypertension (%)	3386 (38.8)	720 (24.8)	1196 (41.1)	1470 (50.6)	**<.001**
Blood glucose status (%)					**<.001**
Diabetes	1326 (15.2)	154 (5.3)	363 (12.5)	809 (27.8)	
Prediabetes	2163 (24.8)	473 (16.3)	789 (27.1)	901 (31.0)	
Normal	5231 (60.0)	2280 (78.4)	1755 (60.4)	1196 (41.2)	
CVD (%)	783 (9.0)	157 (5.4)	279 (9.6)	347 (11.9)	**<.001**
Cardiovascular mortality (%)	414 (4.7)	78 (2.7)	146 (5.0)	190 (6.5)	**<.001**
All-cause mortality (%)	1457 (16.7)	302 (10.4)	522 (18.0)	633 (21.8)	**<.001**

Data are expressed as the mean ± SD, median (upper and lower quartiles), or number (%). Bold indicates *P*-value < .05.

BMI = body mass index, CTI = C-reactive protein-triglyceride glucose index, CVD = cardiovascular disease, FPG = fasting plasma glucose, HDL-C = high-density lipoprotein cholesterol, HOMA-IR = homeostatic model assessment of insulin resistance, LDL-C = low-density lipoprotein cholesterol, SD = standard deviation, TC = total cholesterol, TG = triglycerides, TyG = triglyceride-glucose index.

### 3.2. CVD risk assessment

Multivariable logistic regression analysis revealed a significant association between CTI levels and CVD risk. In the unadjusted model, the highest CTI tertile demonstrated a 2.99-fold increased risk of CVD compared to the lowest tertile (95% confidence interval [CI]: 2.39–3.74; *P* < .001). After adjusting for demographic and lifestyle factors (education, marital status, smoking, alcohol use, and BMI), the association weakened but remained significant (odds ratio [OR] = 1.47, 95% CI: 1.13–1.91; *P* = .013). Further adjustment for clinical variables (LDL-C, hypertension) maintained a similar effect size (OR = 1.50, 95% CI: 1.18–1.92; *P* = .004; Table 2).

**Table 2:** Logistic regression analysis of CTI and CVD risk.

	OR (95% CI)			*P* for trend
	T1	T2	T3	
Model 1	1.00 (Ref)	2.13 (1.66–2.72)	2.99 (2.39–3.74)	<.001
Model 2	1.00 (Ref)	1.17 (0.89–1.53)	1.47 (1.13–1.91)	.013
Model 3	1.00 (Ref)	1.21 (0.93–1.58)	1.50 (1.18–1.92)	.004

Model 1: unadjusted. Model 2: age, sex, race, education, marital status, smoking, alcohol, and BMI. Model 3: model 2 + LDL-C and hypertension.

CI = confidence interval, CTI = C-reactive protein-triglyceride glucose index, CVD = cardiovascular disease, LDL-C, low-density lipoprotein cholesterol, OR = odds ratio.

### 3.3. Association between CTI and mortality risks

**Figure 2.**
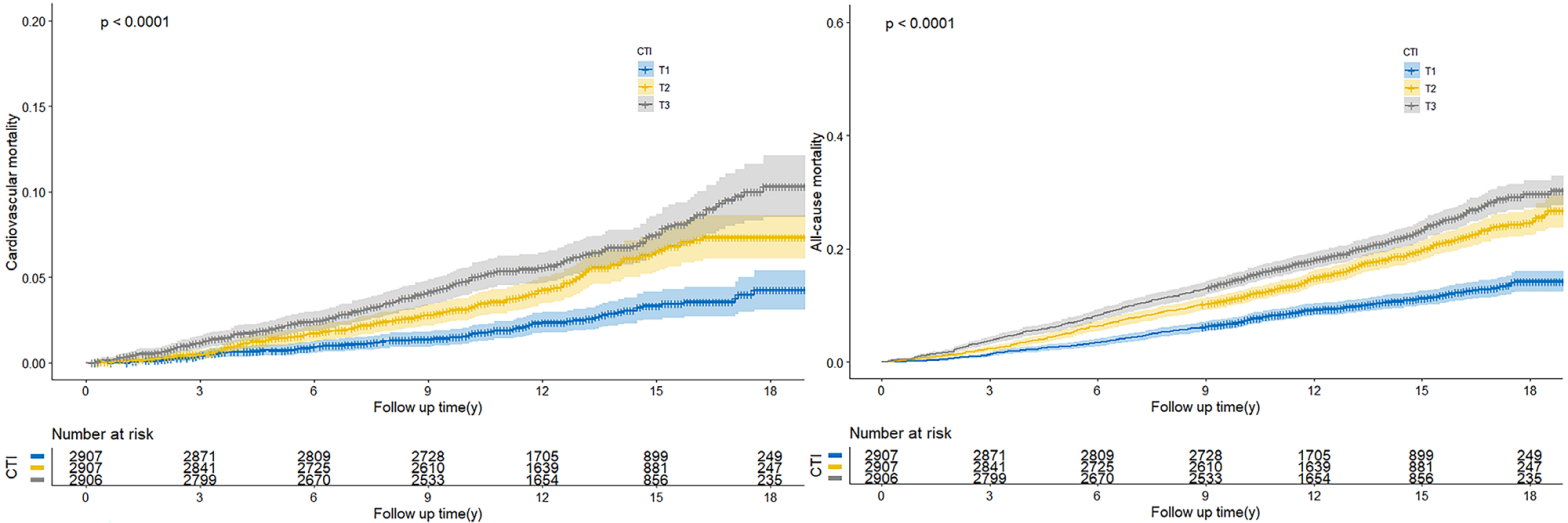
Cumulative survival analysis of cardiovascular and all-cause mortality by CTI tertiles. Survival probabilities are shown over time (units on the *x*-axis) for participants stratified by CTI tertiles (T1, T2, T3). The number at risk at each time point is displayed below the plot. CTI = C-reactive protein-triglyceride glucose index.

The average follow-up time was 12.7 years. Kaplan–Meier analysis demonstrated significantly poorer survival outcomes in the highest CTI tertile (T3) for both cardiovascular and all-cause mortality (log-rank *P* < .0001; Fig. 2). In multivariable Cox regression analyses, the crude model revealed a pronounced cardiovascular mortality risk gradient, with T3 participants exhibiting 3.13-fold higher risk than T1 (95% CI: 2.22–4.40, *P* < .001). This association progressively attenuated with sequential adjustments: first for demographic and lifestyle factors (hazard ratio [HR] = 1.57, 95% CI: 1.07–2.32, *P* = .021), then for LDL-C and hypertension (HR = 1.54, 95% CI: 1.06–2.25, *P* = .024). For all-cause mortality, the highest CTI tertile showed significantly increased risk in all models: unadjusted (HR = 2.69, 95% CI: 2.29–3.15, *P* < .001), after demographic/lifestyle adjustment (HR = 1.52, 95% CI: 1.28–1.81, *P* < .001), following LDL-C/hypertension adjustment (HR = 1.53, 95% CI: 1.29–1.89, *P* < .001; Table 3).

**Table 3:** CTI and mortality risk: a Cox proportional hazards model analysis with sequential adjustment.

Model	HR (95% CI)	*P*-value
Cardiovascular mortality		
Model 1		
T1	1.00 (Ref)	
T2	2.07 (1.45–2.94)	<.001
T3	3.13 (2.22–4.40)	<.001
Model 2		
T1	1.00 (Ref)	
T2	1.04 (0.74–1.45)	.837
T3	1.57 (1.07–2.32)	.021
Model 3		
T1	1.00 (Ref)	
T2	1.03 (0.74–1.44)	.862
T3	1.54 (1.06–2.25)	.024
All-cause mortality		
Model 1		
T1	1.00 (Ref)	
T2	2.00 (1.65–2.44)	<.001
T3	2.69 (2.29–3.15)	<.001
Model 2		
T1	1.00 (Ref)	
T2	1.10 (0.92–1.33)	.302
T3	1.52 (1.28–1.81)	<.001
Model 3		
T1	1.00 (Ref)	
T2	1.11 (0.93–1.33)	.240
T3	1.53 (1.29–1.89)	<.001

Model 1: unadjusted. Model 2: age, sex, race, education, marital status, smoking, alcohol, and BMI. Model 3: model 2 + LDL-C and hypertension.

BMI = body mass index, CTI = C-reactive protein-triglyceride glucose index, LDL-C, low-density lipoprotein cholesterol.

### 3.4. Nonlinear associations

**Figure 3.**
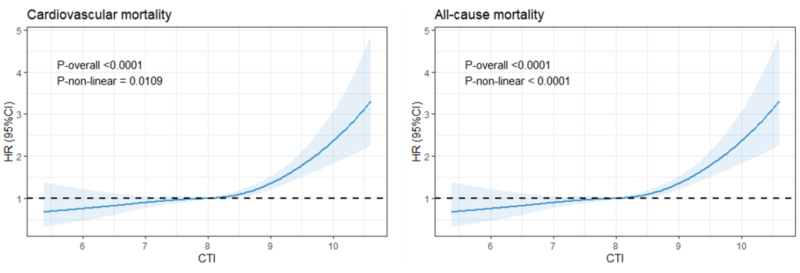
Nonlinear dose–response relationship between CTI and mortality. HRs (95% CIs) for cardiovascular mortality and all-cause mortality increase with rising CTI values. CI = confidence interval, CTI = C-reactive protein-triglyceride glucose index, HR = hazard ratio.

Restricted cubic spline analyses revealed a significant nonlinear relationship between CTI levels and cardiovascular and all-cause mortality risk (*P*-nonlinear < .05), with a steeper increase in mortality risk observed at higher CTI values (Fig. 3).

### 3.5. Sensitivity analysis

To assess the robustness of our findings, we compared baseline characteristics between the retained group (n = 8720) and the excluded group (n = 18,521). Given the large total sample size (>27,000), even minor differences could yield significant *P*-values; therefore, we focused on absolute differences. Most differences between the 2 groups were small: median age differed by 3 years, FPG by 0.11 mmol/L, GHB was identical (5.40 mmol/L), BMI was nearly identical (*P* = .86), and sex (*P* = .113) and alcohol use (*P* = .061) showed no statistical differences. Although some differences in lipid profiles were statistically significant, given substantial missing data in fasting samples, the exclusion criteria did not introduce meaningful selection bias (Table S1, Supplemental Digital Content).

## 4. Discussion

This study demonstrates the associations between CTI and CVD risk (OR = 1.50, 95% CI: 1.18–1.92, *P* = .004), cardiovascular mortality (HR = 1.54, 95% CI: 1.06–2.25, *P* = .024), and all-cause mortality (HR = 1.53, 95% CI: 1.29–1.89, *P* < .001). Previous studies have established the TyG index as a significant predictor of CVD. Studies have shown that higher TyG index values are associated with increased risk of cardiovascular events in patients with coronary artery disease^[12]^ and in the general population.^[13]^ The TyG index has shown utility in predicting CVD events, with higher quartiles linked to greater risk. It may also mediate the effects of other risk factors like body mass index on CVD development.^[14]^ However, the relationship between TyG index and mortality in CVD patients appears to be U-shaped, with both very low and very high levels associated with increased risk.^[15]^

Inflammation is another critical risk factor for CVD. Multiple studies have demonstrated a consistent association between elevated serum CRP levels and increased CVD risk.^[16,17]^ Research suggests that CRP may be an independent predictor of adverse cardiovascular events and mortality.^[16,18]^ Furthermore, CRP levels above 10 mg/L correlate with a higher risk of developing fatal CVD within 10 years.^[17]^

Compared to CRP and the TyG index, the CTI integrates both inflammation and insulin resistance. The CTI was first proposed by Ruan et al as a prognostic marker for poor outcomes in cancer patients.^[19]^

Several studies have demonstrated its clinical relevance in metabolic disorders. For instance, elevated CTI levels have been associated with stroke risk in individuals with dysglycemia and hypertension.^[20,21]^ A NHANES-based study involving 759 patients with comorbid CHD and type 2 diabetes revealed that each 1-unit increase in CTI was significantly associated with a 68% higher risk of all-cause mortality and a 70% increased risk of cardiovascular mortality.^[22]^ Furthermore, a 2025 study from Fuwai Hospital demonstrated that in post-PCI patients, the CTI outperformed the TyG index in predicting recurrent cardiovascular events.^[10]^ A study of 5432 adults without CVD demonstrated that the TyG index independently predicted cardiovascular mortality and all-cause mortality.^[23]^ The findings of this study are partially similar with previous research. In contrast, our study, based on a general population, suggests that CTI’s predictive value for broader cardiovascular outcomes (CHD, myocardial infarction, heart failure) may be necessary.

## 5. Limitations of the study

First, the observational design cannot exclude residual confounding. Second, findings from a general population may not extend to high-risk patients (e.g., those with established coronary artery disease or diabetes). Third, dynamic changes in CTI were not assessed; future studies should explore the prognostic value of CTI monitoring over time.

## 6. Conclusion

Higher CTI is associated with prevalent CVD and independently predicts increased cardiovascular and all-cause mortality.

## Acknowledgments

The authors acknowledge the CDC and the NCHS for providing the NHANES data. We also extend our gratitude to all the participants of the NHANES program and the staff whose efforts made the survey possible.

## Author contributions

**Conceptualization:** Yalei Han.

**Data curation:** Zhenze Yu.

**Formal analysis:** Zhenze Yu.

**Investigation:** MingZhuang Sun.

**Resources:** MingZhuang Sun.

**Software:** Zihan Zhao.

**Supervision:** Yalei Han.

**Writing – original draft:** Zhenze Yu.

**Writing – review & editing:** Yalei Han.


