# Supplementary material for: Association of the C-reactive protein-triglyceride glucose index with cardiovascular disease and mortality in the general US population: A NHANES study
Source: Medicine (Baltimore). 2026 Jul 10;105(28):e49586. doi: 10.1097/MD.0000000000049586 (PMC13363090; doi:10.1097/MD.0000000000049586)
Supplement: Supplementary file 1 [file medi-105-e49586-s001.docx]

Supplementary table

|  | participants | Exclude participants | p-value |
| --- | --- | --- | --- |
| n | 8720 | 18521 |  |
| Age (median [IQR]) | 46.00 [33.00,62.00] | 49.00[34.00,66.00] | <0.001 |
| Sex (male %) | 4245 (48.7) | 8824 (47.6) | 0.113 |
| Race (%) |  |  | <0.001 |
| Mexican American | 1830 (21.0) | 3368 (18.2) |  |
| Other Hispanic | 631 (7.2) | 1192 ( 6.4) | |
| Non-Hispanic White | 4256 (48.8) | 9341 (50.4) | |
| Non-Hispanic Black | 1654 (19.0) | 3783 (20.4) | |
| Other Race - Including Multi-Racial | 349 (4.0) | 837 ( 4.5) | |
| Education (%) | |  | <0.001 |
| Middle school or lower | 2451 (28.1) | 5571 (30.2) |  |
| High school | 2068 (23.7) | 4451 (24.1) | |
| College or more | 4201 (48.2) | 8438 (45.7) | |
| Marital (%) |  |  | 0.01 |
| Married | 4777 (54.8) | 9780 (52.9) |  |
| Divorce | 822 (9.4) | 1867 (10.1) | |
| Other | 3121 (35.8) | 6850 (37.0) | |
| FPG(mmol/L) | 5.44 [5.05,5.94] | 5.55 [5.10, 6.33] | <0.001 |
| GHB | 5.40 [5.20, 5.70] | 5.40 [5.20, 5.80] | <0.001 |
| TC(mg/dl) | 195.00 [169.00,223.00] | 197.00 [170.00, 225.00] | <0.001 |
| LDL-C(mg/dl) | 115.00 [93.00,139.00] | 111.00 [89.00, 135.00] | <0.001 |
| HDL-C(mg/dl) | 52.00 [43.00,64.00] | 50.00 [41.00, 62.00] | <0.001 |
| TG(mg/dl) | 111.00 [79.00,162.00] | 128.00 [86.00, 197.00] | <0.001 |
| BMI(kg/m^2^) | 27.73 [24.28,31.88] | 27.71 [24.16, 32.00] | 0.86 |
| Smoke (%) | 4057(46.5) | 8847 (47.8) | 0.044 |
| Alcohol (%) | 6166 (70.7) | 10325 (69.5) | 0.061 |
| Hypertension (%) | 3386 (38.8) | 7637 (45.5) | <0.001 |
| Blood glucose status (%) |  |  | <0.001 |
| Diabetes | 1326 (15.2) | 2873 (15.5) |  |
| Prediabetes | 2163 (24.8) | 3292 (17.8) |  |
| Normal | 5231 (60.0) | 12333 (66.7) |  |
| CVD (%) | 783 (9.0) | 2301 (12.4) | <0.001 |
| Cardiovascular_mortality (%) | 414 (4.7) | 1066 ( 5.8) | <0.001 |
| All-cause mortality(%) | 1457 (16.7) | 4356 (23.6) | <0.001 |
